# Toward Comprehensive Patient-Centric Care by Integrating Digital Health Technology With Direct Clinical Contact in Australia

**DOI:** 10.2196/12382

**Published:** 2019-06-04

**Authors:** Penelope Schofield, Tim Shaw, Michaela Pascoe

**Affiliations:** 1 Department of Psychology Swinburne University Melbourne Australia; 2 Department of Cancer Experiences Research Peter MacCallum Cancer Centre Melbourne Australia; 3 Sir Peter MacCallum Department of Oncology The University of Melbourne Melbourne Australia; 4 Iverson Health Innovation Research Institute Swinburne University Melbourne Australia; 5 Charles Perkins Centre University of Sydney Sydney Australia; 6 The Institute for Health and Sport Victoria University Melbourne Australia

**Keywords:** health care, health, eHealth

## Abstract

**Background:**

There is an escalating crisis in health care, locally and internationally. The current health care model is unable to meet the increasing health care demands.

**Objective:**

The aim of this study was to reconceptualize the provision of health care to produce better outcomes at no greater cost, by placing individuals in the position of authority to direct their own care, in a personalized, integrated health care system.

**Methods:**

In this study, we used the Australian health care system as a model. We reviewed the current landscape of digital health in Australia and discussed how electronic medical records (EMRs) can be further developed into a personalized, integrated health care system.

**Results:**

Some components of an EMR and digital health system are already being used in Australia, but the systems are not linked. A personalized, integrated health care model that is responsive to consumer needs requires not just a passive repository of medical information; it would require a team approach, including the government, health care funders, industries, consumers and advocacy groups, health care professionals, community groups, and universities.

**Conclusions:**

Implementation of a personalized, integrated health care system can result in reduced pressure on the current health care system, and it can result in the delivery of best-practice health care, regardless of location. Importantly, a personalized, integrated health care system could serve as an education platform, “upskilling” not only clinicians but also, more importantly, patients and carers by providing them with accurate information about their condition, treatment options, medications, and management strategies. By proposing personalized, integrated health care, we offer an intelligent model of health care that is ubiquitous, efficient, and continuously improving.

## Introduction

### Background

Ongoing improvements in health care, successful management of chronic conditions, and falling birth rates in developed nations are globally leading to an aging population [1]. In 1950, 1 person in every 12 people was aged above 60 years [2], by 2015, this had risen to 1 person in every 8 people worldwide [3]. The proportion of older persons is projected to continue to increase, with 1 in 5 people estimated to be aged above 60 by 2050 [3]. The associated prevalence of chronic diseases is immense [1]. By 2020, noncommunicable diseases are expected to contribute to 57% of the global burden of disease and 75% of deaths [4]. Indeed, approximately 40 million people died because of a noncommunicable disease in 2015, namely cardiovascular disease, cancer, chronic respiratory disease, and diabetes, accounting for 70% of deaths worldwide [5]. The societal burden of managing this growing health crisis is substantial and unable to be supported by current models of health care. Like many other countries, Australia is turning to digital technologies to address the rapidly growing gap between service demand and capacity. Achieving a sustainable, agile, and effective health system that keeps pace with demands requires a fundamental disruption of health care delivery. The health system will need to shift focus away from health care providers in centralized locations treating illness; instead, the health system will need to shift focus toward supporting empowered consumers in the community in managing their own health and well-being.

In Australia, the gross domestic product expenditure on health care has increased from 6.5% in 1989-90 to 10.3% in 2015-16 [6,7]. Annually, in Australia, there are an estimated 230,000 medication-related hospital admissions, with a cost to taxpayers of Aus $1.2 billion [8]. The Australian Medicare system is a publicly funded, national universal health care system. In the 2016-17 financial year, Australian Medicare expenditure on medications was Aus $12 million, an increase of 11.3% from the previous financial year [9]. This economic burden is exacerbated by inefficiencies and waste in health care provision. Bentley et al (2008) [10] outlined 4 key inefficiencies: (1) duplication of services, such as repeated blood tests, as clinicians have no access to results of tests ordered by other clinicians; (2) inefficient processes, such as unnecessary transport of patients to seek medical management not available locally; (3) overly expensive inputs, such as physicians providing services that nurses are equally competent to provide; (4) medical errors, such as adverse medication events resulting in avoidable hospital admissions.

The downstream consequence of increasing demand on the health system, errors, inefficiency, and waste is clinician burnout. Like many countries, Australia has a workforce shortage across a number of health professions [11]; therefore, clinicians are struggling to meet the health care requirements of patients [12]. Exacerbating this situation, consumers have increased expectations of clinicians to assist patients in becoming more informed and involved in the treatment decision making and health management and provide prompt, appropriate, and individualized support [12]. Unsurprisingly, high rates of burnout and psychological distress are experienced by medical doctors: indeed, 48% of younger doctors report burnout in the domain of emotional exhaustion, 18% report low professional efficacy, and 46% report high cynicism, according to a survey of over 12,000 Australian medical doctors [13]. Clinician burnout not only greatly reduces quality of life for clinicians but also reduces quality of care to patients and contributes to workforce shortages [14].

The current political climate in health care is characterized by fiscal restraints combined with an aging population [3], increases in the incidence of chronic illnesses [4], and escalating health care costs [6]. These issues are aggravated by a limited and burnt-out workforce trying to meet growing needs [11,14] and significant inefficacies and waste [10]. The existing system of health care is an insatiable beast, which we need to change. Internationally, efforts are presently focused on translating paper-based records into electronic records. In many respects, this represents a digitization of eighteenth century health care and misses a crucial opportunity for technology to transform care and support new disruptive models of care [15]. Currently, routine digital technology use in health is fragmented and mostly “passive” in assisting with information storage or analysis. Although technology heavy, health care has undertaken a digital transformation in the way that businesses, particularly financial services, have. To ensure sustainable effective health care into the future, it is crucial that we implement a digitally supported, fully integrated, and secure health care system that is proactive in the following: tailoring precision, holistic health solutions for the individual; improving equity and access, particularly among the disadvantaged; disseminating health innovations rapidly and upskilling health providers in these innovations; empowering consumers to manage their own health and well-being [16].

### Objectives

The aim of this paper was to reconceptualize the provision of health care to produce better outcomes at no greater cost, by placing individuals in the position of authority to direct their own care. The fundamental premise is that individuals are the experts in their illness experience and personal values in relation to the optimal care they require. In this paper, we use the Australian health care system as a model. We first introduce the concept of a “Bespoke Health Care System” (BHS) and discuss how digital health is currently being implemented in the Australian health care system, including the use and potential of electronic medical records (EMRs). We then propose that EMRs could be integrated into a patient-centered management platform rather than just function as a linked passive information depository. Finally, the necessary conditions for the successful adoption of bespoke health care are discussed, including codesign to develop a platform for the user to ensure positive user experience and trust in the data security and the highlight the importance of utilizing frameworks to identify the barriers most relevant to the implementation BHS.

### Bespoke Health Care

Modern education has adopted an effective new approach of “flipping the classroom,” whereby application of knowledge is done in the classroom whereas didactic learning is self-directed and occurs before class [17,18]. A number of health care practitioners are applying “flipping” to clinical health care, that is, providing patients with necessary knowledge before consultations and then using consultation time to problem solve and make joint decisions, with the aim of improving health outcomes and experience of care and reducing costs [19].

We propose a new health care system that builds upon the flipped health care model to place consumers in the driver’s seat of their health care, termed the BHS. A central component of this model is increased patient involvement in health care decisions and self-management assisted by the use of technology. In the proposed system, individuals will have an EMR, which contains all of a person’s relevant personal and medical information across providers. A total of 1 in 5 medical errors are because of incomplete patient medication information [20]. This record could be linked to primary care clinics, specialists’ rooms, public and private hospitals, pathology services, pharmacies, insurance companies, and other relevant health agencies. It would allow new and relevant medical, diagnostic, and management information to be uploaded and viewed by the individual’s network of health care providers in real time. Where appropriate, individuals would have access to their medical records via the EMR by using their computers and smartphones.

However, the BHS expands well beyond the use of traditional EMRs. Importantly, an EMR need not be a passive depository of data. It could be used to remotely track symptoms, provide routine medication reminders, prompt patients to have a routine screening test, such as a breast mammogram, or allow patients to book medical appointments and remind them to attend. Currently, patient records are still often paper based. Even when they are electronic, they are often not linked, disorganized, and unable to be easily accessed by patients or their wider network of health service providers. This leads to unnecessary reordering of tests, incomplete medical histories, or medication information resulting in suboptimal patient care, occasionally endangering the patient’s life [10].

Importantly, the BHS could serve as an education platform, “upskilling” patients by providing them with accurate information about their condition, treatment options, medications, and self-management strategies. In addition, if patients are prompted to record their clinical data (such as blood sugar levels or blood pressure), medication adherence, or side effects, these data could be aggregated and viewed by the patients and their treatment team to track the illness trajectory and management over time. Moreover, routine symptom assessment could trigger automatically dispatched symptom management advice in real time, suggest appropriate support services available locally, permit clinician notification when a patient’s tracked symptoms indicated the need to review, or prompt the patients to contact their doctor or emergency services if the symptom is potentially life threatening.

The BHS could include digitally supported platforms or ecosystems that integrate care by linking not only electronic records but also patients’ apps and devices with their health record, as well as providing virtual therapeutic spaces for clinicians and patients to interact. Examples of such systems include the Synergy platform, used in treatment of mental health and prevention of self-harm by identifying and rapidly responding to suicidal ideation among young people [21]. Through “upskilling” individuals, this initiative would empower people to have greater ownership over the management of their health. Finally, the BHS could be used to also provide clinicians with point-of-care decision support by providing diagnostic algorithms to be used in conjunction with clinical assessments and up-to-date, evidence-based, and easily accessible optimal care pathways suitable for their patient’s condition, which will also serve as continuing professional development. Thus, although an EMR is the core of the BHS, there are a number of other equally important components required to implement the BHS.

### The Current Landscape of Digital Health in Australia

Some components of an EMR system are already being used in Australia [22]. EMRs are commonly used in primary care, and they are being introduced into hospital settings [23,24]. Several large scale Australian hospitals have fully implemented an EMR and are using the system to electronically share prescription and medication details directly with pharmacy, eliminating written scripts, which avoids errors associated with translating handwriting [22]. This results in a safer and more efficient medication management [24]. Importantly, this system gives health care professionals more detailed access to information about medications taken, improving informed clinical decision making, which is particularly beneficial for individuals with comorbid conditions who are taking multiple medications [23,24]. These digital hospitals are also using EMRs to provide electronic orders to diagnostic providers, such as radiology and pathology within the hospital setting [23,24]. Some other areas where an EMR could provide significant benefits include improved safety and health care quality and greater patient, clinician, and administrator satisfaction, as well as cost savings and revenue gains, such as through reduced drug expenditure [25].

In Australia, this technology is being rapidly adopted by hospitals around the country (see Table 1), which presents some examples of technologies being adopted by Australian hospitals and health care practices.

Australia’s “My Health Record” was launched in July 2012, and it is a patient-controlled, secure Web-based summary of health information, which can be accessed by individuals by using their computer or smartphones. This initiative will greatly assist Australia in achieving an integrated digital health system. Patients can control what clinical information is uploaded and select to share this information with their primary care physician, specialists, hospitals, and other health care providers. According to Australia’s National Digital Health strategy, by 2022, the “My Health Record” system will allow clinicians to share patient information with other health care providers, using a nationally consistent, standards-based approach to secure messaging [30].

**Table 1:** Examples of digital health technologies adopted by Australian hospitals and health care practices.

Location	Digital health technology
South Australia	The state of South Australia has introduced an “Enterprise Patient Administration System,” which is an integrated EMR^a^ system for every patient admitted to a public hospital or health service, progressively being rolled out across all metropolitan public hospitals and a network of general practices in South Australia [26].
Tasmania	The Tasmanian state government has implemented a real-time reporting and recording system of controlled drugs. This system allows prescribers and pharmacists to determine what Schedule 8 drugs have been dispensed for a patient, if a patient has had his or her access to drugs of dependence restricted, or if a patient has been declared to be drug dependent or drug seeking by a medical practitioner [27].
Australia wide	Australia’s “My Health Record” is a patient-controlled, secure Web-based summary of health information, which can be accessed by individuals by using their computer or smartphones.
The Northern Territory	The Northern Territory of Australia, which is geographically very remote and lacks many specialist health services, routinely provides telehealth services, which have increased appointment attendance and reduced patient travel time and expenses [28].
Queensland	The long-term strategy for the state of Queensland is a Digital Hospital and Health Service [29]; therefore, the government’s next intended step is to link the EMR among care settings across the health system [29]. The Queensland state health department has already invested in the development of an integrated EMR across 9 facilities and 7 hospital and health services, with the aim of enabling the exchange of information among primary, community, and acute care settings across the health system [29]. In Queensland [30], all public hospitals and health services are already connected to the “My Health Record” system, and a number of hospitals and health services are connected in other parts of Australia [31].
Victoria	In the state of Victoria, several health services have implemented an electronic referral process, so that referrals are sent electronically from 1 health care service to another rather than via fax or post [32].

^a^EMR: electronic medical record.

Furthermore, individuals and health care providers will have access to information about prescribed and dispensed medicines, which will limit abuse of prescription medications, such as opioids [33,34]. Patients will be able to digitally request their medicine on the Web, and all pharmacists will have access to electronic prescribing [30,35]. The National Digital Health Strategy is also prioritizing an end-of-life care pilot test to explore how advanced care planning documents can be incorporated into the “My Health Record,” to be more accessible to treating health professionals [30]. In addition, the “My Health Record” provides a data platform for new interfaces to be added onto the system, and a number of apps are currently authorized to connect the “My Health Record” system, including “Health Engine,” which connects individuals to health practitioners and allows Web-based booking of appointments [36]. The Australian government has developed a Web-based mental health portal that provides information about mental health apps and services. “My Aged Care” is a similar portal where individuals can access information on aged care and related care services.

In the pilot scheme before 2018, about 20% of Australians registered for a “My Health Record” [37]. This scheme was mandated nationally from mid-2018, with a record being automatically created for all Australians, unless they “opted out” [38-41]. Currently, 90% of Australians have a “My Health Record” [42]. According to Australia’s National Digital Health strategy, the “My Health Record” is set to be the core component of Australia’s future national digital health service [30]. In Queensland, [30] all public hospitals and health services are already connected to the “My Health Record” system, and a number of hospitals and health services are connected in other parts of Australia [31]. In Australia alone, the potential economic benefit of transitioning to an EMR system in all public acute and private hospitals equates to approximately Aus $1.76 billion annually [25]. This would be far greater if expanded to community and primary care settings [25]. Telehealth is an important part of digital health and a BHS. Indeed, Mate and Salinas (2014) [19] highlighted that many elements of face-to-face clinic visits can be performed at home, with the aid of modern technology, which is consistent with and important for the development of a BHS, particularly for those individuals who lack access to specialist care because of geographical isolation or limited mobility [43]. It has been estimated that in Australia, telehealth can not only improve access to medical care and increase convenience for patients but it may also reduce cost by up to AU $3 billion annually through reduced residential care costs, emergency admissions, potentially preventable hospitalizations, and Royal Flying Doctors Services in rural areas, as well as patient transport and travel [44,45]. Currently, the Australian Federal and State Governments are expanding digital health to improve connectivity among service providers in community and primary care settings and reach people in their own homes [25]. Teleconference consultations between clinicians and individuals in remote areas and living in residential care facilities are currently being conducted as part of a home monitoring study and funded through the Australian Medicare system [44,45]. To facilitate telehealth services, a local telecommunications company, Telstra, has developed the “My Care Manager,” a tablet-based platform where clinicians, individuals, and carers can video conference, where medication details, scheduled services, and test reminders and care plans can be stored, and where data from medical devices, such as body weight, glucose, and pulse oximeters can be uploaded and remotely monitored by clinicians [46]. If part of an integrated BHS, it is possible that additional medical requirements could be done remotely or more locally, for example, medication reviews could be done virtually [47], and individuals could have blood draws taken at local centers [48,49]. Currently, national agencies in Australia have tested a system to remotely monitor vital signs, such as electrocardiography, heart rate, spirometry, noninvasive blood pressure, oxygen saturation, body weight, glucometry, and body temperature, in a cohort of elderly individuals with chronic illnesses [45,50]. Finally, a number of technology providers in Australia, such as Springday [51] or InnoWell [52] are providing integrated platforms that provide support for new models of care that integrate devices, apps, and virtual consultations into one-stop consumer-facing platforms in the management of chronic diseases, such as mental health or cancer [21]. These initiatives demonstrate the potential of an integrated BHS, which could integrate EMRs, remote access to medical care, and consultation and health tracking, among other functions. In summary, an integrated digital health system is evolving in Australia to allow individuals to access test results, set up appointments, medication reminders, test reminders, and have teleconsultations with health care providers, but routine implementation of these systems is patchy, and the systems that are in place are often not linked. It is yet to be articulated how this new model of health care would operate on a population level.

## Bespoke Health Care System: Moving Electronic Medical Records From an Information Depository to a Patient-Driven Management Platform

We argue that an intelligent approach using electronic personal medical records to place individuals at the center of their care and “upskill” them to have ownership over the management of their health care will lead to greater efficiencies and better patient outcomes. Indeed, individuals have expressed a desire to be empowered. The Australian Digital Health Agency led the extensive “Your health. Your say” consultation to inform the development of the Australia’s National Digital Health strategy. More than 3000 consumers, carers, health care providers, community groups, professional bodies, and other stakeholders attended 103 forums, workshops, webcasts, and town hall meetings across Australia, and over 1000 submissions and survey responses were analyzed for key themes [30]. The first identified theme was that individuals want to take control of their health care decisions and need access to their personal health information to do this [30]. Participants identified that they wanted to be able to manage their medication, request prescription refills, and track their health status by using their smartphone [30,35]. An integrated BHS would allow individuals to track symptoms, have access to diagnostic test results and treatment decision aids, and obtain reliable information about their diagnoses, management plans and medications, and self-management strategies for disease, symptom, or side-effect management. In addition, these systems would support the integration of apps and devices, health data, and virtual care to support new, potentially disruptive models of care. Previous research shows that it is possible to increase self-management of care through technology [53]. For example, an electronic health diary and symptom tracker/health monitoring tool have been shown to improve self-efficacy in individuals with acute coronary syndrome [54]. This proposal mimics the flipped classroom model, whereby individuals can engage in didactic learning at home [17,18,55], and their time with clinicians can therefore be focused on discussing and solving problems and applying the knowledge learned earlier [55]. The BHS could also be used to assist individuals to adhere to clinical interventions. Indeed, electronic prompts and reminder alerts have been shown to increase patient engagement with digital interventions [56]. Symptom monitoring and patient feedback through the BHS could be used to assign patients to the appropriate level of stepped care, and the provision of care tailored to the individual’s level of need could be delivered using Web-based interventions through the system. Piette et al [57] have developed a personalized cognitive behavior therapy pain management service that adapts to each patient’s unique and changing needs to automatically personalize the intensity and type of patient support, using feedback from patients about their progress. Technology would enable the tailoring to individual, clinical, and personal circumstances by using reinforcement learning algorithms to deliver information that is most relevant to each user, similar to those algorithms used by Netflix, Google, and Amazon [58]. This system could also involve and “upskill” family/carers, which would increase the capacity of the health care system by expanding nonprofessional community care. Research shows that an interactive Web portal providing targeted support for informal caregivers of persons with dementia and professionals improves the acquisition of information, interactions between carers and professionals, access to support from home, and self-perceived empowerment in health-related decisions [59]. A further advantage of the BHS is that it could be used to link interested individuals to peer support networks, which could result in greater social connectedness and support and improved coping strategies [60,61]. Individuals could “opt in” to be linked with other individuals with similar diagnoses and circumstances. Finally, this system would enable consumer ownership of the individuals’ health data, increasing consumer health literacy, engaging consumers in treatment decision making, activating health promoting behaviors and self-management, and supporting consumers to remain in their homes andlocal communities. Such a system would place consumers in the “driver’s seat” in regard to the management of their own care. The increasing focus on self-management of chronic disease in many respects places the clinician in the position of coach rather than director of health care, much in the same way education has shifted from a model of the all-knowing expert who lectures to students to a model focused on cocreation of learning, where the teacher guides rather than directs learning through a collaborative environment. Textbox 1 displays an imaginary case study of how the BHS may operate from the patient’s perspective.

**Imaginary case study of how the Bespoke Health Care System may operate from the patient’s perspective.**

*Georgia is in her late 70s and lives at home with her husband Frank and their 2 corgis, Lucy and Roxy. Georgia has diabetes, which is generally well controlled. Georgia’s smartphone beeps at her and notifies her that she is due to meet with her General Practitioner, Susan, for a regular checkup. Georgia and her General Practitioner are both connected to a Bespoke health care platform that enables them to communicate with one another, as well as other health care professionals, through secure messaging and web-based face-to-face meetings. It is this platform that has notified Georgia that she is due to meet with Susan.*

*Using the Bespoke health care platform on her smartphone, Georgia sees that Susan has a free appointment time available on Thursday morning, which Georgia books. The Bespoke health care platform also notifies Georgia that she is due to have some routine blood tests as part of her checkup, and it shows her some available times at the pathology clinic around the corner from Georgia’s house. There is a spot available tomorrow afternoon, and Georgia selects that time. She will take Lucy and Roxy with her on her walk to the pathology clinic.*

*After her appointment, the pathology clinic uploads Georgia’s test results to her Bespoke health care platform. This allows Susan, Georgia’s General Practitioner, to see her results instantly, and if appropriate, to make these available for Georgia to view as well. Fortunately, all of Georgia’s tests results are normal, and Susan sends Georgia a message telling her this.*

*On Thursday, Georgia and Susan have a web-based face-to-face appointment using the Bespoke health care platform. The platform securely records their meetings. At any time, Georgia and Susan both go back and review anything that they discussed during their appointment.*

*After her appointment, Georgia realizes that she is running low on her medication. She logs in to her Bespoke health care platform and requests a refill of her regular medication, the details of which are already stored in her profile. Susan remotely approves this request, and it is automatically sent to the pharmacy of Georgia’s choosing. Georgia selects the pharmacy closest to her house. The pharmacist then makes the medication ready for Georgia to pick up, and the pharmacist notifies her using the Bespoke health care platform.*

*On Saturday morning, Georgia and Frank decide to take Lucy and Roxy for walk to the pharmacy to collect Georgia’s medication. They will also visit their favourite café for breakfast beforehand. Unfortunately, after collecting her medication, Georgia rolls her ankle and has a small fall. She is alright, but she needs to go to the hospital for some monitoring. At the hospital, Georgia’s treating physician, Alex, is able to view Georgia’s medical history, medication information, and treatment plan, as well as any allergies or other relevant medical information, using the Bespoke health care platform. All of the information about Georgia’s visit to the hospital, including her treatment plan, is also uploaded to the Bespoke health care platform, and Georgia’s General Practitioner, Susan, is notified to check in with Georgia regarding her ankle at their next appointment.*

*On Sunday afternoon, Georgia is settling in back at home, and The Bespoke health care platform automatically sends her some practical tips about caring for her ankle and a reminder to take the medication that she collected from the pharmacy yesterday, as per Susan’s recommendations, and it’s a good thing it did, as Georgia was preoccupied thinking about her ankle.*

### Necessary Conditions for Successful Adoption of Bespoke Health Care

The critical element to achieve a BHS is the establishment of a universal personalized EMR, which has already occurred with the “My Health Record [30]” in Australia. All individuals would need to have access to fast, reliable, ubiquitous internet, and there would need to be widespread ownership and proficient use of mobile devices. Community and specialist involvement and infrastructure would be necessary. A BHS would require the involvement of local community centers to provide education in the BHS, host peer support groups, deliver coaching in self-management, and possibly use motivational interviewing techniques to increase engagement with the system [62]. The system would need to be underpinned by best available evidence, regularly updated, and have high acceptability with health care providers, patients, carers, and privacy advocates. The BHS would also present new opportunities for digitally supported communication between health providers and patients to support innovative models of care. Critically, universal health records need to be able to securely store data from such emerging systems. Potential benefits of the BHS are multiple. First and foremost, the system would place the consumers at the center of their care and promote self-management. Individuals living in regional/remote areas would have greater access to peak health services and specialist clinicians via teleconsultations. The application of optimal care pathways could be offered, regardless of the patient’s location, which would reduce regional variations in health outcomes [1]. Patients would have access to real-time, evidence-based self-management advice. There would be reduced duplication and errors. Moreover, clinical encounters would be optimized by automating routine clinical activities, such as assessment of symptoms or side effects. Furthermore, more clinical time could be devoted to health care decision making and education, such as reinforce health promoting behaviors. The intent is that the BHS would enable the routine and universal delivery of best-evidence health care, resulting in optimal health outcomes and better patient experiences of the health system. The system should demonstrate improved or equivalent patient outcomes, with a reduction or no increase in health care costs. Finally, iterative and quality improvement processes based on performance data could be built into the BHS. The development of this system requires a team approach. On a government level, there would need to be regulation and oversight, standards, systems, and infrastructure to support the BHS. Health care funders, such as Australia’s Medicare and insurance agencies, would need to change the architecture of their funding models, which currently center around face-to-face medical consultations with an individual patient and the delivery of procedures. Progress is being made in this domain, with teleconsultations between clinicians and individuals in remote areas and Residential Care Facilities, which are currently being funded through the Medicare system [44,45]. Industries would need to develop and roll out new business and care models based around personalized medicine and informed by best-evidence practice. Universities can play a key role in training future health professionals’ in digital health care systems, as well as supporting the analysis and interpretation of big data, research, evaluation, and iterative improvement of the BHS. The needs of consumers and advocacy group end users would need to be identified and integrated, as was done in the “Your health. Your say” consultation [30]. The experiences of clinicians working within the health system must also be considered and integrated through processes, such as codesign models.

To be accepted and utilized by patients and health professionals, the initiative must be designed to be acceptable to the end users (both patients and health professionals), cater to individuals’ unique needs to place minimal demands on the health system infrastructure, and be scalable and rapidly disseminated into usual care if successful. Co-design is crucial to the intervention development process to design for the user. Co-design seeks to understand the lived experience of end users, making the everyday practices and contexts of the target audience important resources to inform the design of the intervention [63]. Co-design refers to collective creativity as it is applied across the whole span of a design process [64]. Specifically, this entails experts (researchers, designers, or developers) working together with end users (consumers) from concept creation, prototype review, to final product. In addition, theoretical frameworks should be considered to provide the basis to optimize implementation strategies and to identify implementation barriers, strategies to address these barriers and methods to explore mediating mechanisms [65]. A comprehensive understanding of the barriers and enablers to implementation of a BHS in clinical care is necessary to support the development of effective implementation strategies [66,67]. Dansereau and colleagues outlined that there are 4 levels of analysis relevant to organizational behavior, these are persons, dyads, groups, and organizations [68-70]. Evidence shows implementation requires whole system change, involving both the individual and organization [71]. Theoretical frameworks can help explain why implementation efforts succeed or fail [72], but the biggest challenge influencing the implementation of new interventions is behavior change, with many frameworks therefore focusing on human behavior theories [73,74]. The Theoretical Domains Framework (TDF) developed by Michie and colleagues [74] used expert consensus to identify 12 domains of behavior determinants that could be used in implementation research. Utilizing frameworks similar to TDF will be useful in identifying the individual- and organizational-level barriers most relevant to the implementation of a BHS. Consumer ownership of data and privacy considerations also need to be addressed. The Australian Digital Health Agency acknowledged this concern in the National Digital Health strategy, and it has established the Digital Health Cyber Security Centre. Its primary purpose is to protect the national digital health systems and personal health information of Australians from cyber threat and raise the security posture of the Australian health sector. The Digital Health Cyber Security Centre partners with national and international cyber security organizations, across the government and private sector, to improve knowledge of the cyber threat and leverage shared expertise and material across organizations [30]. The recent funding of the Digital Health Cooperative Research Centre by the Australian Federal Government that links academia with services, government, and industry also provides a vehicle for the development and support of new national, coordinated approaches to the development of new technology-driven models of care [75].

## Conclusions

The ultimate objectives of the proposed BHS are reducing pressure on the current health care system, delivering best-practice health care, regardless of location, and placing consumers in a position to direct their own health care. Delivery of personalized, evidence-based health care, regardless of location, is likely to improve disease outcomes, quality of life, and patient experiences. “Upskilling” individuals and enabling easy information sharing can improve communication between individuals and health care providers. The BHS can offer real-time, accurate data collection and information dissemination. Importantly, delivery of best practice health care in regional locations would aid in decentralizing health services by stimulating health industry growth in regional centers, as well as reducing the pressure and resource constraints in urban hospitals. This system would also contribute to the development of a next generation workforce, which is responsive to emerging challenges, particularly in the digital domain. By proposing BHS, we offer an intelligent model of health care that is ubiquitous, efficient, and continuously improving. The system would allow research and iterative improvements to be embedded into health systems. Finally, reducing service production and delivery waste is likely to result in economic benefits.

## Abbreviations

BHS: Bespoke Health Care System
EMR: electronic medical record
TDF: Theoretical Domains Framework
